# The First Complete Mitochondrial Genome of Common Hedge Blue *Acytolepis puspa* (Lepidoptera: Lycaenidae), and Comparative Genomic Analysis Within Polyommatinae

**DOI:** 10.1002/ece3.73326

**Published:** 2026-03-29

**Authors:** Muyang Li, Dan Zhang, Jianping Zhang, Dongkai Liu, Xianfeng Yi, Ran Li

**Affiliations:** ^1^ School of Life Sciences Qufu Normal University Qufu China; ^2^ Characteristic Laboratory of Forensic Science in Universities of Shandong Province Shandong University of Political Science and Law Jinan China

**Keywords:** comparative analysis, Lepidoptera, mitochondrial genome, phylogeny, Polyommatinae

## Abstract

The butterfly genus *Acytolepis* (Lepidoptera: Lycaenidae: Polyommatinae) is widely distributed in the Indomalayan and Australasian realms. However, no complete mitochondrial genome has been reported for this genus, leaving its mitogenomic characteristics and evolutionary patterns unclear. In this study, we sequenced and characterized the first complete mitogenome of *Acytolepis puspa*, the type species of the genus. The circular double‐stranded mitogenome is 15,511 bp in length and comprises 13 protein‐coding genes (PCGs), two ribosomal RNA genes (rRNAs), 22 transfer RNA genes (tRNAs), and one A + T‐rich control region, exhibiting a typical gene content and organization conserved in Lepidoptera. Comparative analyses of Polyommatinae mitogenomes revealed pronounced heterogeneity in evolutionary rates among PCGs, with *ND6* and *ND3* showing relatively high nucleotide diversity, whereas *COX1* was the most conserved gene. Selection pressure analyses indicated that all PCGs are evolving under purifying selection, with *ATP8* and *ND6* exhibiting relatively relaxed selective constraints compared to other genes. Phylogenetic analyses based on concatenated mitochondrial PCGs using both Maximum likelihood (ML) and Bayesian inference (BI) methods produced identical and well‐supported topologies, recovering *A. puspa* as the sister taxon to *Celastrina* species within Polyommatinae. Overall, this study provides the first mitogenomic resource for *Acytolepis*, enriches mitochondrial molecular markers for lycaenid butterflies, and contributes new insights into mitogenome evolution and phylogenetic relationships within Polyommatinae.

## Graphical abstract

This study firstly reports the complete mitochondrial genome of Acytolepis puspa, providing an important mitogenomic reference for the previously unrepresented genus Acytolepis.

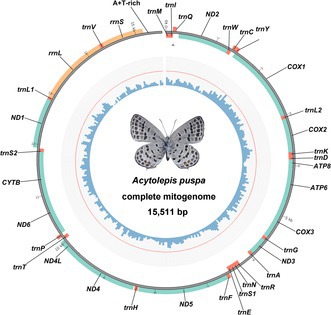

## 1 Introduction

The insect mitochondrial genome (mitogenome) is typically a closed, circular, double‐stranded DNA molecule ranging from 14 to 20 kb in length. It contains a highly conserved set of 37 genes: 13 protein‐coding genes (PCGs), two ribosomal RNA (rRNA) genes, 22 transfer RNA (tRNA) genes, and a non‐coding A + T‐rich region that encompasses essential regulatory signals for replication and transcription (Wolstenholme 1992; Boore 1999; Cameron 2014). Due to its compact size, maternal inheritance, low recombination, relatively high mutation rate, and stable gene content, the mitogenome has become an important molecular marker for species identification, phylogeographic inference, evolutionary genomics, and systematic studies in insects (Lou et al. 2018; Yan et al. 2021; Zhou and Yang 2022; Elyasigorji et al. 2023; Ge et al. 2025). Notably, phylogenetic analyses based on complete mitogenomes often yield more robust and reliable results than those derived from single mitochondrial loci, providing enhanced resolution across both shallow and deep evolutionary timescales (Li et al. 2020; Ge et al. 2023).

Lepidoptera, the second‐largest order of insects, comprises nearly 160,000 described species across 137 families and 43 superfamilies worldwide (Weng et al. 2025). Lepidopterans occupy diverse ecological niches and play integral roles in terrestrial ecosystems as herbivores, pollinators, and prey species (Ghazanfar et al. 2016; Macrì et al. 2023). Many taxa are economically important, functioning as major agricultural and forestry pests, ornamental species, or classical model organisms for studies on development, mimicry, genetics, and environmental adaptation (Takov et al. 2020). Despite extensive research, establishing a stable and universally accepted classification for this highly diverse order remains challenging, and earlier phylogenetic hypotheses were predominantly based on morphological characters (Queiroz‐Santos et al. 2018).

With the rapid development of next‐generation sequencing (NGS), the availability of complete mitogenomes has increased markedly, accelerating the use of mitogenomic data in Lepidoptera systematics (Abbasi 2025). Mitogenome‐based analyses have contributed to resolving taxonomic relationships across multiple hierarchical levels, including studies within Hesperiidae, Uraniidae, Saturniidae, and other major families and superfamilies (Xue et al. 2024; Cai and Yin 2025; Balakrishnan et al. 2025). Although the number of sequenced lepidopteran mitogenomes is growing, current records in GenBank still represent less than 1% of known species and encompass only a subset of lepidopteran superfamilies and families, highlighting substantial taxonomic gaps and the need for broader sampling (Majeed et al. 2025). Expanding mitogenome coverage across genera and subfamilies is therefore crucial for improving phylogenetic resolution and understanding evolutionary diversification in Lepidoptera.

*Acytolepis* is a small genus within the subfamily Polyommatinae (Lycaenidae), originally described by Lambertus Johannes Toxopeus (Stradomsky 2016). It currently includes five recognized species distributed throughout the Indomalayan and Australasian realms (Chandrasekharan and Palot 2020). Despite its broad distribution and taxonomic significance, no complete mitogenome has been reported for any species of *Acytolepis*. Consequently, key aspects of its mitogenomic architecture, evolutionary features, and phylogenetic placement within Polyommatinae remain unknown. Comparative mitogenomic analyses within Polyommatinae offer an opportunity to elucidate lineage diversification, evolutionary rates, and taxonomic boundaries within this speciose lycaenid subfamily.

In this study, we sequenced and annotated the first complete mitogenome of *Acytolepis puspa*, the type species of the genus, using high‐throughput sequencing technology. We characterized its genomic structure, including gene content, gene arrangement, codon‐usage patterns, nucleotide composition, and features of the A + T‐rich region. Comparative analyses were performed using mitogenomes of ten additional Polyommatinae species to assess conserved and variable genomic traits. Furthermore, phylogenetic relationships within Lycaenidae were reconstructed using both Maximum Likelihood (ML) and Bayesian Inference (BI) methods. This study provides the first mitogenomic resource for *Acytolepis* and offers new insights into the molecular evolution and phylogenetic relationships of Polyommatinae. This work lays a cornerstone for future comparative genomics and phylogenetic studies aimed at resolving complex evolutionary relationships within Lepidoptera.

## 2 Materials and Methods

### 2.1 Sample Collection and DNA Extraction

Adults of *A. puspa* were collected from Hechi, Guangxi Zhuang Autonomous Region, China. In accordance with relevant national legislation and institutional guidelines, no specific permission was required for the collection of the insect specimen used in this study, as it is not an endangered or protected species. The specimens were immediately preserved in anhydrous ethanol and stored at −20°C for subsequent DNA extraction. Species identification was performed based on the morphological descriptions provided by Yuan et al. (2015). All voucher specimens were deposited in the School of Life Sciences, Qufu Normal University.

Genomic DNA was extracted from thoracic muscle tissue using the Takara Universal Genomic DNA Extraction Kit (Takara, Japan) following the manufacturer's instructions. DNA integrity was examined by 1% agarose‐gel electrophoresis, and purity and concentration were measured using a NanoDrop 2000 spectrophotometer (Thermo Scientific, Wilmington, USA). High‐quality DNA was aliquoted into sterile microtubes and stored at −20°C for subsequent sequencing.

### 2.2 Mitogenome Sequencing and Assembly

Qualified genomic DNA was sent to Biozeron Biotechnology Co. Ltd. (Shanghai, China) for library construction and sequencing on the Illumina NovaSeq 6000 platform. Libraries were prepared using the TruSeq DNA PCR‐Free HT Kit (Illumina, San Diego, USA), and paired‐end 150 bp reads were generated, yielding approximately 5 Gb of raw data.

Low‐quality reads and adapter sequences were filtered out using NGS QC Toolkit v2.3.3 (Patel and Jain 2012), and data quality was assessed with FastQC v0.11.9 (Brown et al. 2017). High‐quality clean reads were subjected to de novo assembly in NOVOPlasty v4.0 (Dierckxsens et al. 2017), with the *COX1* gene fragment of *A. puspa* (accession number: ON999056) used as a seed sequence.

### 2.3 Mitogenome Annotation and Analysis

The assembled mitogenome was initially annotated using the online MITOS server (http://mitos.bioinf.uni‐leipzig.de/index.py) under the invertebrate mitochondrial genetic code (Bernt et al. 2013). Protein‐coding genes (PCGs) were further checked and manually corrected by aligning with homologous sequences from published Lycaenidae mitogenomes. Intergenic spacer regions and overlapping sequences were identified manually in MEGA X (Kumar et al. 2018). Transfer RNA secondary structures were predicted using tRNAscan‐SE v1.21 and visualized with RNAstructure v5.4 (Chan et al. 2021; Reuter and Mathews 2010). Repeat motifs within the control region were identified with Tandem Repeats Finder (Benson 1999). A circular map of the mitogenome was generated using MitoZ (Meng et al. 2019).

Ten available Polyommatinae mitogenomes were retrieved from GenBank for comparative analyses (Table 1). Base composition, codon usage, and relative synonymous codon usage (RSCU) were calculated using MEGA X and PhyloSuite v1.2.1 (Zhang et al. 2020). Nucleotide skewness was computed as AT‐skew = (A − T)/(A + T) and GC‐skew = (G − C)/(G + C). Nucleotide diversity (Pi) of the 13 PCGs across Polyommatinae was estimated in DnaSP v6 using a sliding window of 100 bp and a step size of 25 bp (Rozas et al. 2017). Non‐synonymous (Ka) and synonymous (Ks) substitution rates and Ka/Ks ratios were also calculated in DnaSP v6 to evaluate selective pressures acting on PCGs.

**TABLE 1:** Information of the mitogenomes used for the phylogenetic analysis in this study.

Family	Subfamily	Species	Size (bp)	Accession number
Lycaenidae	Polyommatinae	*Acytolepis puspa*	15,511	This study
		*Celastrina oreas*	15,216	ON964515
		*Celastrina lavendularis*	15,221	ON920451
		*Cupido argiades*	15,330	KC310728
		*Caerulea coeligena*	15,164	MZ489120
		*Jamides bochus*	15,537	OM948811
		*Agriades orbitulus*	15,410	ON868909
		*Polyommatus amorata*	15,389	ON411620
		*Glaucopsyche lygdamus xerces*	15,252	MW677564
		*Plebejus argus*	15,426	MN974526
		*Shijimiaeoides divina*	15,259	KT897723
	Theclinae	*Coreana raphaelis*	15,314	DQ102703
		*Protantigius superans*	15,248	HQ184265
		*Hypaurotis quercus*	15,366	KM592971
		*Japonica lutea*	15,225	KM655768
	Lycaeninae	*Lycaena phlaeas*	15,280	JX262887
	Aphnaeinae	*Spindasis takanonis*	15,349	HQ184266
	Curetinae	*Curetis bulis*	15,162	JX262888
		*Curetis acuta*	15,178	MZ196213
Hesperiidae	Coeliadinae	*Burara striata*	15,327	KY524446
		*Choaspes benjaminii*	15,300	KJ629164

### 2.4 Phylogenetic Analysis

Thirteen PCGs were extracted from the mitogenomes of 17 species of Lycaenidae and two species of Hesperiidae as outgroups (Table 1) for phylogenetic inference. Each PCG was aligned separately using the L‐INS‐i strategy in MAFFT v7.205 (Katoh and Standley 2013). Substitution saturation was tested with DAMBE v5 (Xia and Xie 2001), and poorly aligned regions were removed using Gblocks v0.91b under relaxed parameters (Talavera and Castresana 2007). All trimmed alignments were concatenated into a single matrix using PhyloSuite v1.2.1.

The best partitioning scheme and corresponding nucleotide substitution models were selected with ModelFinder based on the corrected Akaike Information Criterion (AICc) (Kalyaanamoorthy et al. 2017). Maximum likelihood (ML) analysis was performed in RAxML v8.2.0 with 1000 bootstrap replicates (Stamatakis 2014). Bayesian inference (BI) was conducted in MrBayes v3.2.2 using two independent Markov chain Monte Carlo (MCMC) runs of 10,000,000 generations, sampling every 1000 generations and discarding the first 25% as burn‐in (Ronquist et al. 2012). Resulting trees were visualized and edited in FigTree v1.4.2 (Rambaut 2019).

## 3 Results and Discussion

### 3.1 Mitogenome Organization and Rearrangement Pattern

**FIGURE 1:**
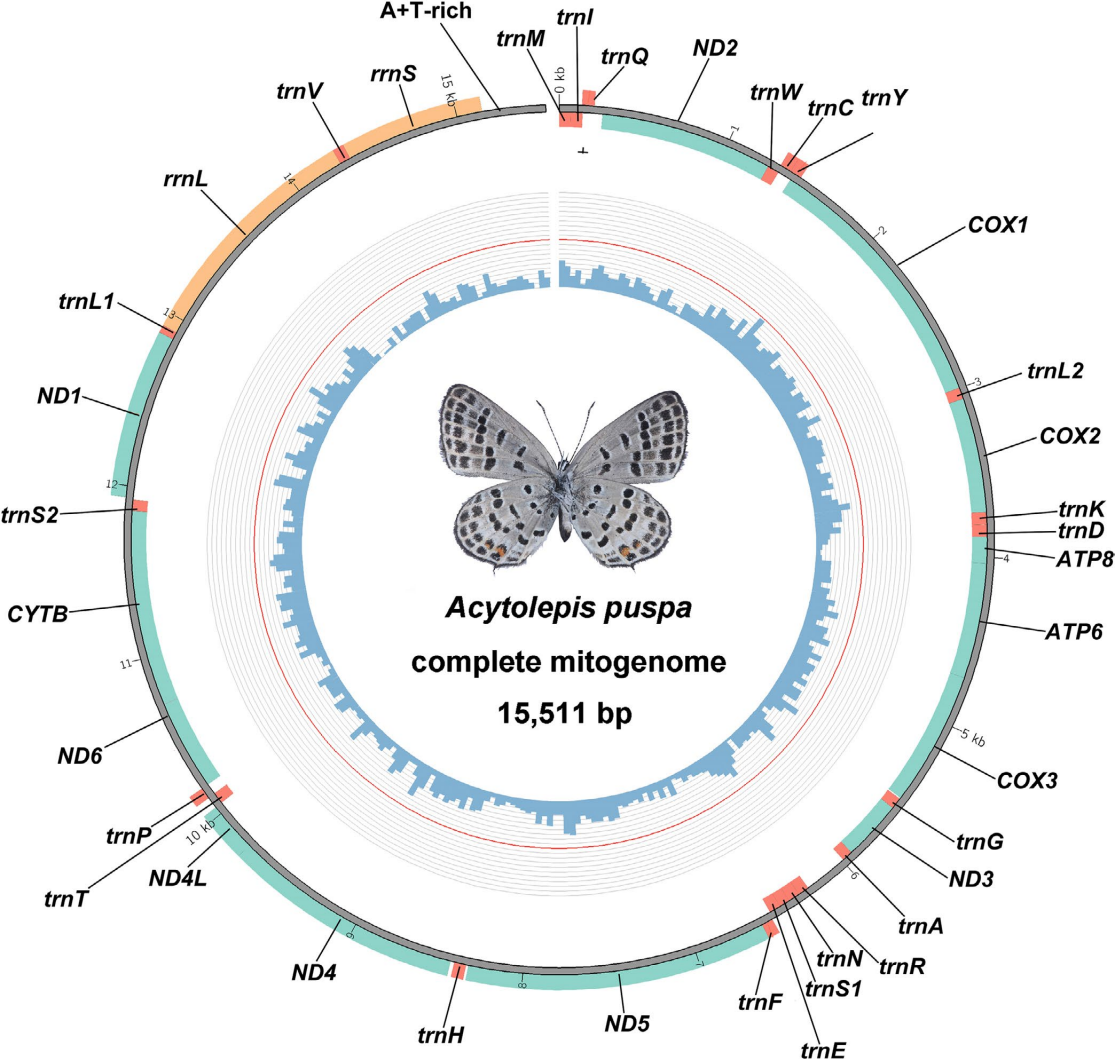
Circular map of the mitogenome of *A. puspa*. External genes are transcribed anticlockwise, and internal genes are clockwise. The internal blue histogram indicates the GC content.

The mitogenome of *A. puspa* is a closed circular, double‐stranded molecule with a total length of 15,511 bp, which falls within the size range reported for Lepidoptera mitogenomes (Gu et al. 2025). The mitogenomic sequence has been deposited in the GenBank database (accession number: PX797440). It encodes the complete and typical metazoan mitochondrial gene complement, including 13 protein‐coding genes (PCGs), 22 transfer RNA genes (tRNAs), two ribosomal RNA genes (rRNAs), and a single non‐coding control region (A + T‐rich region) (Table 2; Figure 1). Gene orientation conforms to the conserved lepidopteran pattern, with 23 genes encoded on the majority (J) strand and the remaining 14 genes located on the minority (N) strand. This strand distribution is highly conserved across Lepidoptera and reflects strong evolutionary constraints on mitochondrial transcriptional organization (Park et al. 2016).

**TABLE 2:** Mitogenome characteristics of *A. puspa*. J and N represent the positive and negative strands of the mitogenome, respectively.

Gene	Strand	Location		Size (bp)	IN	Anticodon	Start codon	Stop codon
		From	To					
*trnM*	J	1	68	68	0	CAU		
*trnI*	J	69	134	66	0	GAU		
*trnQ*	N	132	200	69	−3	UUG		
*ND2*	J	254	1267	1014	53		ATT	TAA
*trnW*	J	1266	1332	67	−2	UCA		
*trnC*	N	1325	1392	68	−8	GCA		
*trnY*	N	1393	1458	66	0	GUA		
*COX1*	J	1465	2995	1531	6		CGA	T‐—
*trnL2*	J	2996	3062	67	0	UAA		
*COX2*	J	3064	3736	673	1		ATG	T‐—
*trnK*	J	3737	3807	71	0	CUU		
*trnD*	J	3811	3880	70	3	GUC		
*ATP8*	J	3881	4042	162	0		ATA	TAA
*ATP6*	J	4036	4713	678	−7		ATG	TAA
*COX3*	J	4713	5501	789	−1		ATG	TAA
*trnG*	J	5513	5578	66	11	UCC		
*ND3*	J	5579	5932	354	0		ATT	TAG
*trnA*	J	5931	5993	63	−2	UGC		
*trnR*	J	6255	6321	67	261	UCG		
*trnN*	J	6321	6389	69	−1	GUU		
*trnS1*	J	6390	6449	60	0	UCU		
*trnE*	J	6450	6515	66	0	UUC		
*trnF*	N	6527	6593	67	11	GAA		
*ND5*	N	6593	8314	1722	−1		ATT	TAA
*trnH*	N	8330	8396	67	15	GUG		
*ND4*	N	8421	9761	1341	24		ATG	TAA
*ND4L*	N	9761	10,048	288	−1		ATG	TAA
*trnT*	J	10,052	10,116	65	3	UGU		
*trnP*	N	10,117	10,181	65	0	UGG		
*ND6*	J	10,184	10,711	528	2		ATA	TAA
*CYTB*	J	10,716	11,861	1146	4		ATG	TAA
*trnS2*	J	11,860	11,923	64	−2	UGA		
*ND1*	N	11,938	12,879	942	14		ATG	TAA
*trnL1*	N	12,881	12,948	68	1	UAG		
*rrnL*	N	12,949	14,283	1335	0			
*trnV*	N	14,284	14,349	66	0	UAC		
*rrnS*	N	14,350	15,145	796	0			
A + T‐rich	J	15,146	15,511	366	0			

Notably, the gene order of *A. puspa* deviates from the ancestral insect mitochondrial arrangement (*Drosophila yakuba*, *trnI‐trnQ‐trnM*) and instead exhibits the characteristic lepidopteran tRNA cluster rearrangement *trnM‐trnI‐trnQ* (Clary and Wolstenholme 1985; Li et al. 2021). This rearrangement has been consistently documented across diverse lepidopteran lineages and is widely regarded as a molecular synapomorphy of the order Lepidoptera (Li et al. 2021). It is generally interpreted as the result of tandem duplication–random loss (TDRL) events that occurred early in lepidopteran evolutionary history (Cameron 2014). The presence of this conserved rearrangement in *A. puspa* further corroborates its placement within Lycaenidae and reinforces the remarkable structural stability of mitogenomes at higher taxonomic levels within Lepidoptera.

### 3.2 Nucleotide Composition and Strand Asymmetry

The mitogenome of *A. puspa* exhibits a pronounced bias toward adenine (A) and thymine (T) nucleotides, with an overall A + T content of 82.04%, which is well within the range reported for lepidopteran mitogenomes and reflects a typical feature of insect mitogenomes (Table S1). Among different genomic regions, the A + T content is lowest in PCGs (80.86%) and highest in the control region (90.43%), a pattern commonly attributed to stronger functional and structural constraints acting on coding regions, in contrast to the relatively relaxed selective pressures on non‐coding sequences.

Analyses of strand asymmetry revealed region‐specific skew patterns, with the complete mitogenome and control region exhibiting negative AT‐skews (−0.011 and −0.021, respectively) and GC‐skews (−0.149 and −0.542, respectively), indicating an excess of T over A and cytosine (C) over guanine (G), respectively (Table S1). In contrast, PCGs exhibit negative AT‐skew (−0.137) but positive GC‐skew (0.052), whereas both tRNA and rRNA genes show positive values for AT‐skew (0.008 and 0.044, respectively) and GC‐skew (0.163 and 0.305, respectively). This heterogeneity in nucleotide skew among different genomic components is a widespread phenomenon in insect mitogenomes (Jiang et al. 2016).

Such strand‐specific compositional asymmetry is generally interpreted as the consequence of asymmetric mutational pressures associated with mitochondrial DNA replication and transcription, particularly the unequal exposure of the leading and lagging strands to mutagenic processes such as deamination (Haag‐Liautard et al. 2008). Similar region‐dependent patterns of nucleotide composition and strand asymmetry have been extensively documented across Lepidoptera and other holometabolous insect orders, underscoring the conserved nature of mitochondrial compositional evolution and the shared mechanistic basis underlying strand bias in insect mitogenomes (Raden et al. 2020).

### 3.3 Protein‐Coding Genes and Codon Usage

The 13 protein‐coding genes (PCGs) of *A. puspa* span a total length of 11,165 bp, accounting for 71.98% of the complete mitogenome (Table S1). Among these genes, *ATP8* is the shortest PCG (162 bp), whereas *ND5* is the longest (1722 bp), a gene‐length distribution that is highly consistent with those reported for other lepidopteran and insect mitogenomes (Chen et al. 2023; Li et al. 2020, 2021). All PCGs initiate with typical mitochondrial start codons, except *COX1*, which employs the alternative start codon CGA. The use of non‐canonical start codons in *COX1* has been widely documented across insects and is now regarded as a conserved feature of insect mitogenomes rather than an annotation anomaly (Zhang et al. 2016). Regarding termination codons, *COX1* and *COX2* end with incomplete single‐nucleotide T stop codons, whereas the remaining 11 PCGs terminate with complete standard stop codons (TAA or TAG). These truncated stop codons are presumed to be converted into functional termination signals through post‐transcriptional polyadenylation, a well‐established mechanism in animal mitochondrial gene expression (Zhang et al. 2025).

**FIGURE 2:**
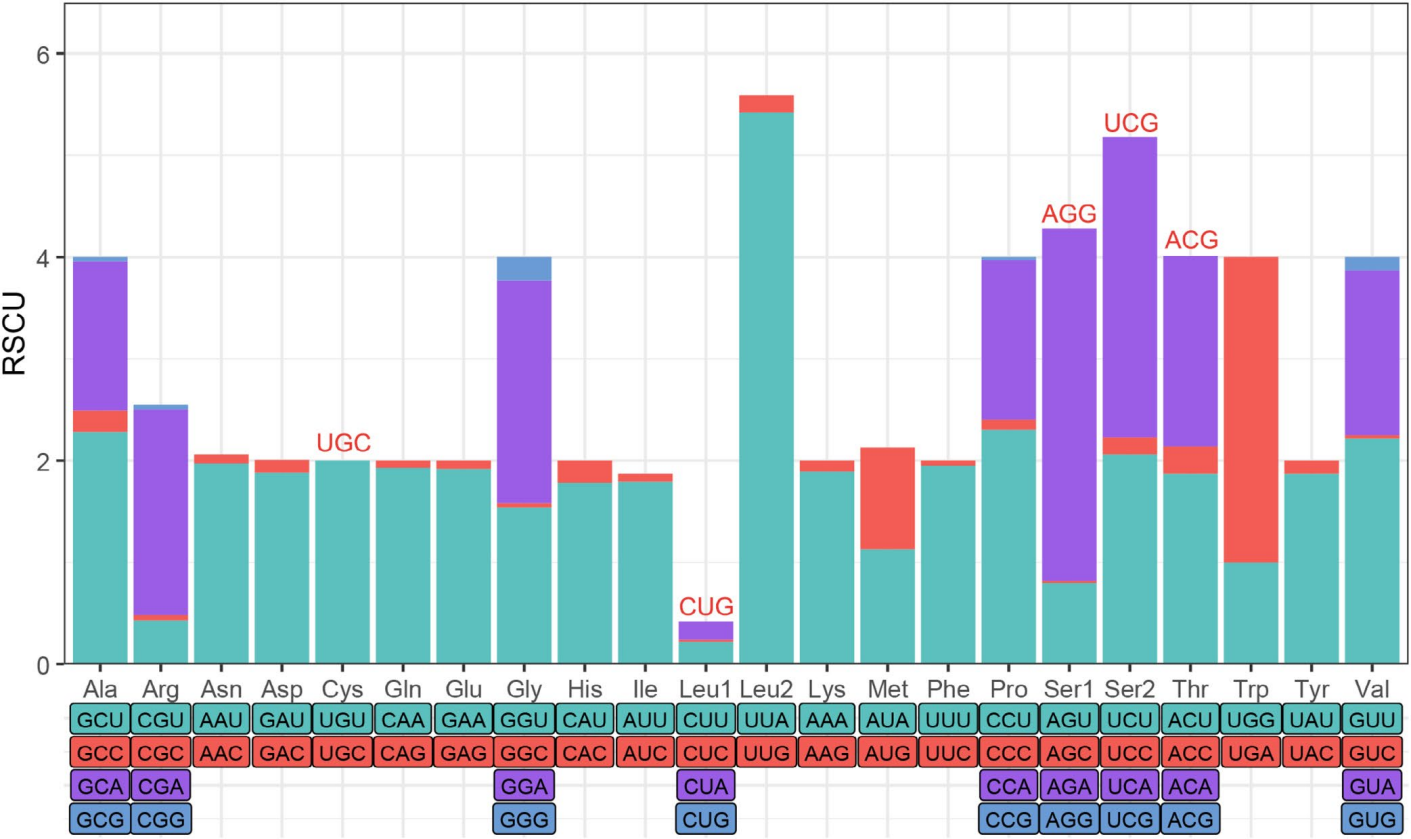
Relative synonymous codon usage (RSCU) of the *A. puspa* mitogenome. The red codons denote the undetected codons in this analysis.

After excluding termination codons, the 13 PCGs of *A. puspa* encode a total of 3711 amino acids. Analysis of relative synonymous codon usage (RSCU) revealed a strong codon usage bias (Figure 2; Table S2). Several GC‐rich codons, including UCG (Ser), UGC (Cys), ACG (Thr), AGG (Ser), and CUG (Leu), were absent, whereas codons with high A or T content were preferentially utilized. In particular, UUA (Leu), AGA (Ser), and UGA (Trp) exhibited the highest RSCU values (5.42, 3.46, and 3.00, respectively), indicating strong selective or mutational preference for these codons. This pronounced codon usage bias is closely associated with the overall A + T‐rich nucleotide composition of the mitogenome and reflects the combined effects of mutational pressure and translational selection. Similar patterns of AT‐driven codon usage bias have been widely reported in Lepidoptera and other insect orders, suggesting that mitochondrial codon usage evolution is largely shaped by compositional constraints rather than adaptive optimization at the amino acid level (Natalia et al. 2025).

### 3.4 Ribosomal and Transfer RNA Genes

The mitogenome of *A. puspa* contains two ribosomal RNA genes, the large subunit rRNA (*rrnL*; 1335 bp) and the small subunit rRNA (*rrnS*; 796 bp) (Table 2). Consistent with the conserved organization of lepidopteran mitogenomes, *rrnL* is located between *trnL1* and *trnV*, whereas *rrnS* is positioned between *trnV* and the control region. Both rRNA genes exhibit relatively high A + T content and display positive AT‐skew and GC‐skew values (Table S1). This compositional skew pattern differs from that reported in many lepidopteran species, suggesting the presence of lineage‐specific variation in rRNA nucleotide composition, potentially associated with differences in replication dynamics or relaxed structural constraints.

A complete set of 22 transfer RNA genes was identified in the *A. puspa* mitogenome, with individual tRNA lengths ranging from 60 to 71 bp (Table 2). Secondary structure predictions indicate that all tRNAs, except *trnS1*, fold into the typical cloverleaf structure (Figure S1). The absence of the dihydrouridine (DHU) arm in *trnS1*, which instead forms a simple loop, is a well‐documented and conserved feature across insect mitogenomes and is generally not considered to compromise tRNA functionality (Yang et al. 2025). Analysis of tRNA secondary structures revealed a total of 26 non‐canonical base pairings, including thirteen G–U wobble pairs, seven U–U mismatches, three A–A mismatches, two A–G mismatches, and one U–C mismatch, in addition to standard Watson–Crick base pairs (A–U and G–C). Such non‐canonical pairings are frequently observed in insect mitochondrial tRNAs and are thought to be stabilized through RNA editing or tertiary structural interactions, thereby maintaining the functional integrity of mitochondrial translation (Kunzmann et al. 1998; Helm et al. 2000).

### 3.5 Non‐Coding Regions

Variations in non‐coding regions, especially in the number and length of overlapping sequences and intergenic spacers, significantly affect mitogenome length across different insects (Lv et al. 2018). In the mitogenome of *A. puspa*, several short intergenic spacers were identified, with the longest spacer (261 bp) located between *trnA* and *trnR* (Table 2). A conserved overlapping sequence between *ATP8* and *ATP6* was also detected. In most lepidopteran species, this region is characterized by a highly conserved 7 bp overlap with the sequence (ATGATAA), whereas an alternative sequence (ATGATAG) has been reported in Micropterigoidea (Chen et al. 2020). In *A. puspa*, the canonical lepidopteran overlapping motif “ATGATAA” was present (Figure S2), further supporting the strong evolutionary conservation of transcriptional and translational coupling between *ATP8* and *ATP6* within Lepidoptera.

**FIGURE 3:**
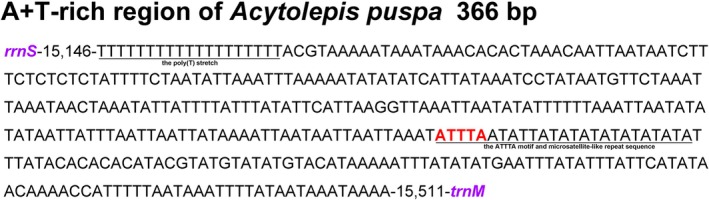
Features present in the A + T‐rich region of the *A. puspa* mitogenome. The poly(T) and the microsatellite‐like repeat sequence adjacent to “ATTTA” are underlined.

The A + T‐rich region is the largest non‐coding region and contains signals that regulate DNA replication and transcription (Fernandez‐Silva et al. 2004). In *A. puspa*, this region spanned 366 bp and possessed the highest A + T content (90.43%) (Table S1). In Lepidoptera mitogenomes, the A + T‐rich region typically contains conserved features including the origin of light‐strand replication (OL), a 5′‐terminal poly(T) stretch, an upstream poly(A) stretch adjacent to *trnM*, a highly variable domain, and a microsatellite‐like repeat sequence (Cameron and Whiting 2008). A 19 bp poly(T) stretch is located at its 5′ end in the A + T‐rich region of the mitogenome of *A. puspa*. The presence of such an extended poly(T) stretch is a conserved feature associated with the mitochondrial origin of replication in Lepidoptera, creating a single‐stranded loop that serves as the recognition and binding site for the replication machinery (Wu et al. 2020) (Figure 3). Microsatellite‐like repeat sequences in the A + T‐rich regions of lepidopterans typically follow the signature motif “ATTTA”. A microsatellite‐like repeat sequence “ATTTA(AT)_2_(TA)_8_” was detected in the *A. puspa* mitogenome, starting at position 15,382 bp (Figure 3).

### 3.6 Comparative Analysis of Polyommatinae Mitogenomes

**FIGURE 4:**
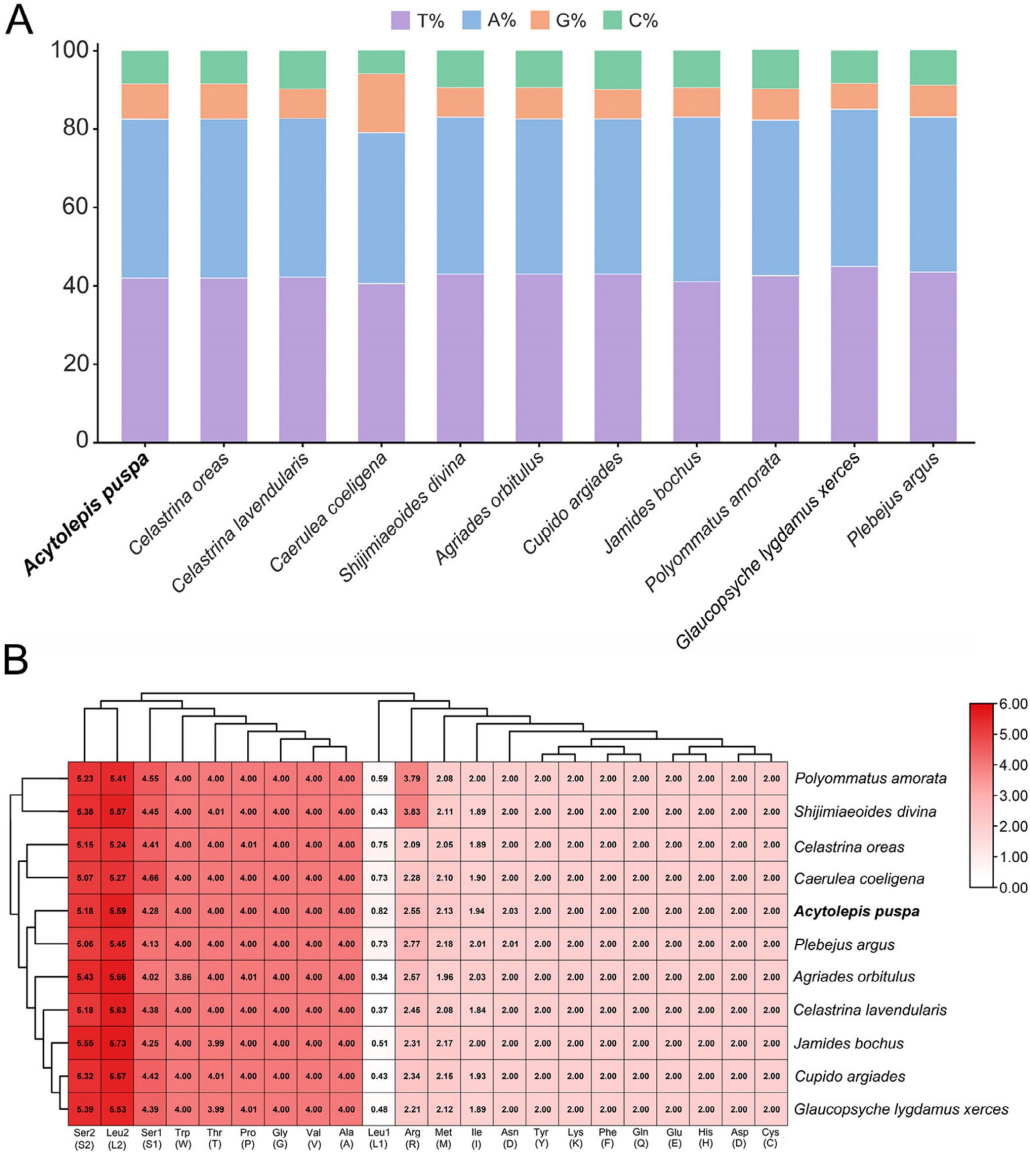
Compositional analysis of mitogenomes in Polyommatinae species. (A) Nucleotide composition, showing the relative frequencies of the four nucleotides (A, T, C, and G); (B) Amino acid composition, illustrating the relative usage frequency of the 20 amino acids in protein‐coding genes.

Comparative analysis of the complete mitogenomes from 11 Polyommatinae species revealed a consistently high A + T content across all taxa (Figure 4A). The lowest A + T content was observed in *Caerulea coeligena* (79.8%), whereas the highest was detected in *Glaucopsyche lygdamus xerces* (82.4%), indicating relatively limited interspecific variation in nucleotide composition within the subfamily. Analysis of amino acid composition across the 13 PCGs showed highly conserved patterns among the 11 Polyommatinae species (Figure 4B). Leucine (Leu) and serine (Ser) were consistently the most frequently encoded amino acids, both predominantly represented by codons with high A or T content. This pronounced amino acid and codon usage bias is closely associated with the overall A + T‐rich nature of Polyommatinae mitogenomes and is generally attributed to long‐term mutational pressure favoring synonymous codons ending in A or T, rather than adaptive changes in protein function (Fernandez‐Silva et al. 2004; Xia 1996).

**FIGURE 5:**
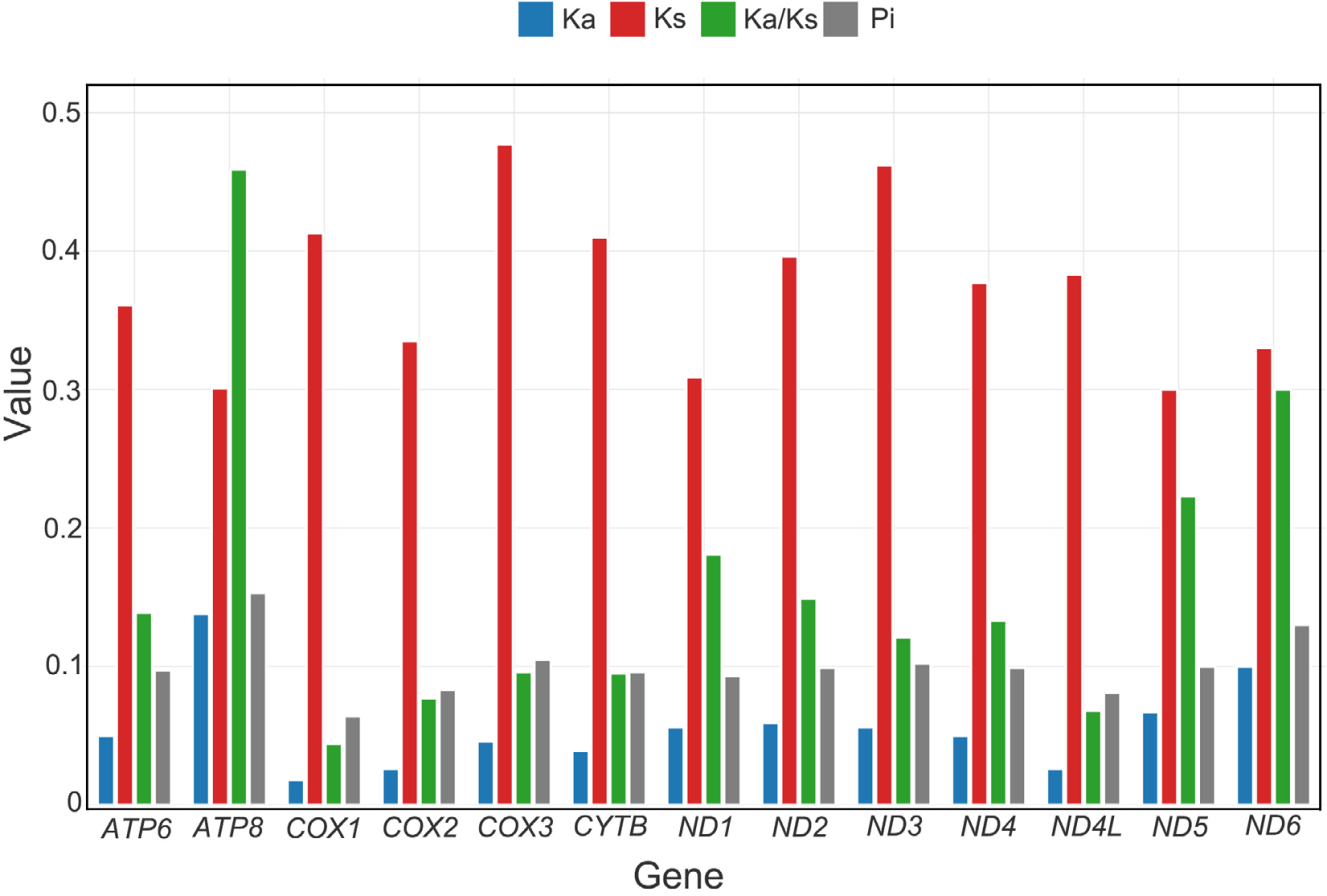
Ka, Ks, Ka/Ks, and Pi values from 13 protein‐coding genes (PCGs) within Polyommatinae. Ka, non‐synonymous mutation rate; Ks, synonymous mutation rate; Ka/Ks, the ratio of non‐synonymous mutation rate to synonymous mutation rate; Pi, nucleotide diversity.

To evaluate selective pressures acting on mitochondrial PCGs, the numbers of non‐synonymous substitutions (Ka), synonymous substitutions (Ks), and their ratios (Ka/Ks) were calculated for all 13 PCGs across the 11 Polyommatinae species (Figure 5). The average Ka/Ks ratios for all genes were substantially below one, indicating that mitochondrial PCGs in Polyommatinae are evolving under purifying selection (Zhang and Li 2004). Among these genes, *COX1*, *COX2*, and *COX3* exhibited particularly low Ka/Ks values, reflecting strong functional constraints associated with their essential roles in oxidative phosphorylation. In contrast, *ATP8* displayed the highest Ka/Ks ratios, followed by *ND6* and *ND5*, suggesting relatively relaxed purifying selection, a pattern that has been repeatedly observed in Lycaenidae and other lepidopteran lineages (Chen et al. 2023).

Nucleotide diversity (Pi) was further estimated for the 13 PCGs to assess patterns of evolutionary variability among Polyommatinae mitogenomes (Figure 5). Sliding window analysis (window size: 100 bp; step size: 25 bp) revealed pronounced heterogeneity in nucleotide diversity across different genes. *ND6* and *ND3* exhibited relatively high Pi values, indicating increased sequence variability during Polyommatinae evolution. In contrast, *COX1* consistently showed the lowest Pi values, confirming its status as the most conserved mitochondrial gene in Lepidoptera (Shi et al. 2024). Notably, although *ATP8* exhibited both the highest Ka/Ks ratios and comparatively high average Pi values, these results should be interpreted with caution. The short length of *ATP8* (typically less than 170 bp) relative to the sliding window size can artificially inflate both nucleotide diversity estimates and Ka/Ks ratios. Thus, the elevated values likely reflect the statistical bias due to short sequence length rather than genuinely heightened evolutionary pressure.

### 3.7 Phylogenetic Relationships

**FIGURE 6:**
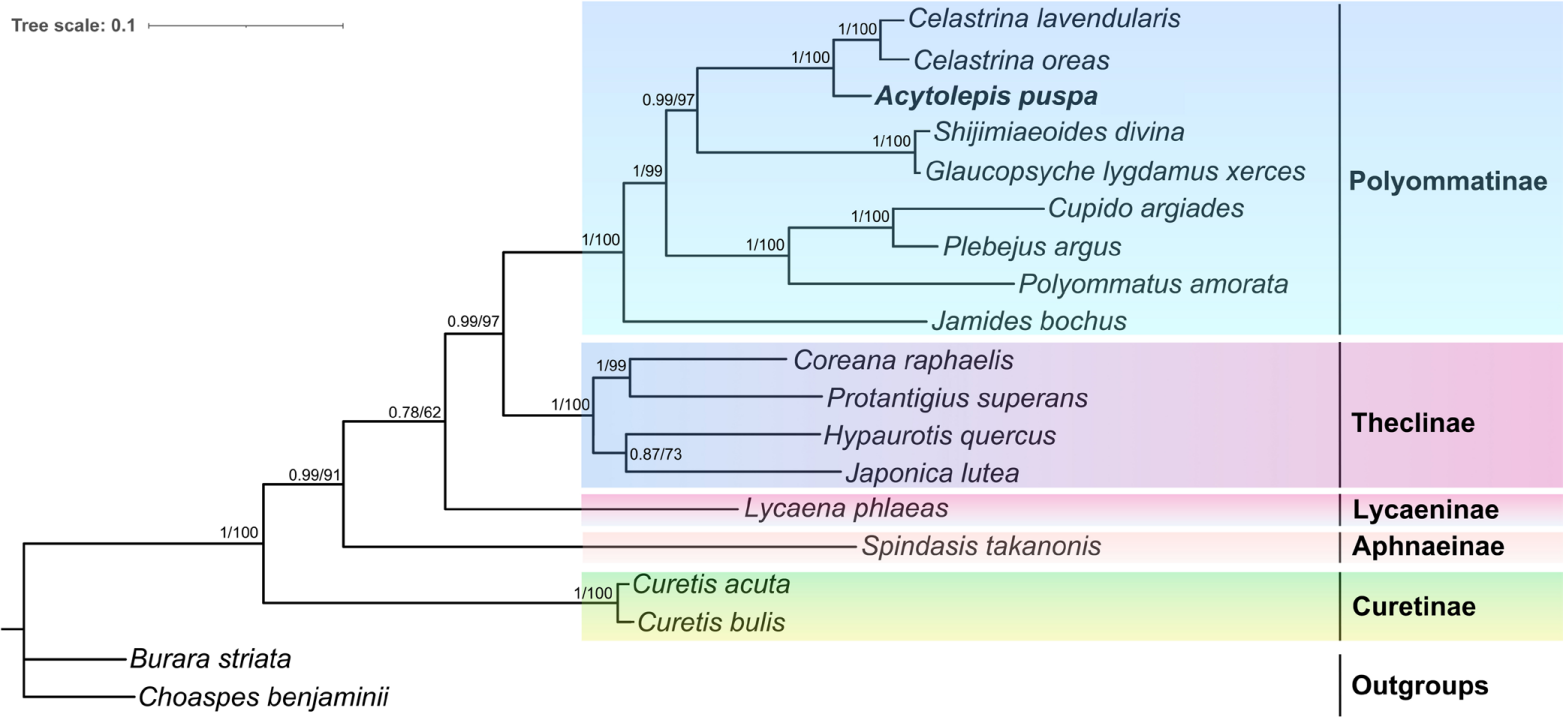
Phylogenetic trees inferred from Maximum likelihood (ML) and Bayesian inference (BI) analyses based on the 13 protein‐coding genes (PCGs). The posterior probability (PP) and bootstrap support value (BS) are shown on the nodes.

Phylogenetic relationships were reconstructed based on a concatenated dataset of 13 PCGs from 19 butterfly species, including 17 representatives of Lycaenidae and two Hesperiidae species used as outgroups. Before tree reconstruction, substitution saturation was assessed using DAMBE, and the results (ISS < ISS.c) confirmed that the dataset retained sufficient phylogenetic signal for reliable inference. Both Maximum Likelihood (ML) and Bayesian Inference (BI) analyses yielded fully congruent topologies, with strong nodal support for most internal branches (Figure 6).

Among the Lycaenidae subfamilies represented by more than one species, Curetinae, Theclinae, and Polyommatinae were each recovered as monophyletic with high support. Owing to the limited availability of complete mitogenomes for Lycaenidae, two additional subfamilies were represented by single taxa, which resulted in reduced support at several deeper nodes. Within Lycaenidae, Curetinae, represented by *Curetis acuta* and *C. bulis*, was robustly recovered as the earliest diverging lineage (PP = 1.00, BS = 100). In addition, Theclinae and Polyommatinae were strongly supported as sister groups (PP = 0.99, BS = 97). These relationships are fully congruent with previous phylogenetic hypotheses based on complete mitogenome data, supporting the stability of higher‐level relationships within Lycaenidae.

Relationships within Polyommatinae were particularly well resolved, with all internal nodes receiving strong statistical support. The analyzed taxa represent eight distinct genera, although only one genus (*Celastrina*) was represented by more than one species. *Jamides bochus* was recovered as the earliest diverging lineage within Polyommatinae. Notably, *A. puspa* formed a strongly supported sister group with the two *Celastrina* species (*C. lavendularis* and *C. oreas* ). This relationship corroborates previous phylogenetic studies based on nuclear and mitochondrial markers (*COX1*, *EF‐1α*, and *ITS2*), which placed *Acytolepis* and *Celastrina* within the subtribe Lycaenopsina and indicated a close evolutionary affinity between the two genera (Stradomsky 2016). Overall, the phylogenetic relationships recovered in this study are consistent with earlier molecular analyses and further demonstrate the utility of mitogenome data for resolving intergeneric relationships within Polyommatinae, despite current limitations in taxon sampling (Chen et al. 2023; Zhou et al. 2020).

## 4 Conclusions

This study reports, for the first time, the complete mitogenome of *Acytolepis puspa*, providing an important mitogenomic reference for the previously unrepresented genus *Acytolepis*. The mitogenome exhibits a typical lepidopteran architecture, with conserved gene content, gene arrangement, and a pronounced A + T nucleotide bias, reflecting the structural stability of mitogenomes within Polyommatinae. Comparative analyses indicate that mitochondrial protein‐coding genes in this subfamily are predominantly constrained by purifying selection, although evolutionary rates inferred from substitution patterns (Ka/Ks ratios and nucleotide diversity) vary among genes. Phylogenetic analyses based on complete mitochondrial protein‐coding genes produced well‐supported and congruent topologies, consistently recovering *A. puspa* as closely related to species of *Celastrina*. These findings highlight the utility of whole mitogenomes as effective molecular markers for resolving phylogenetic relationships within Lycaenidae. Nevertheless, given the limited availability of mitogenomic data and uneven taxon sampling in Polyommatinae, broader sequencing efforts across additional genera and species will be essential to further refine the evolutionary framework of this diverse butterfly group.

## Author Contributions

**Muyang Li:** investigation (equal), methodology (equal), resources (equal), software (equal), validation (equal), writing – original draft (lead), writing – review and editing (equal). **Dan Zhang:** investigation (equal), methodology (equal), software (equal), validation (equal), writing – original draft (equal). **Jianping Zhang:** data curation (equal), investigation (equal), methodology (equal), software (equal), writing – original draft (equal). **Dongkai Liu:** investigation (equal), resources (equal), software (equal), writing – original draft (equal). **Xianfeng Yi:** conceptualization (equal), investigation (equal), project administration (equal), supervision (equal), visualization (equal), writing – review and editing (equal). **Ran Li:** conceptualization (equal), funding acquisition (lead), investigation (equal), project administration (lead), supervision (equal), visualization (equal), writing – original draft (equal), writing – review and editing (equal).

## Funding

This research was funded by the Natural Science Foundation of Shandong Province, grant number ZR2025MS341, and the Youth Innovation Team Development Project of Shandong, grant number 2024KJC010.

## Conflicts of Interest

The authors declare no conflicts of interest.

## Supporting information


**Figure S1:** Predicted secondary structures for the tRNAs of *A. puspa* mitogenome. Red: amino acid acceptor arm; Blue: TψC arm; Aqua green: variable loop; Purple: anticodon arm; Orange: dihydrouridine arm.

**Figure S2:** The conserved overlapping sequences between ATP8 and ATP6 across lepidopterans used in this study.

**Table S1:** Nucleotide composition and AT/GC skew patterns in different regions of *A. puspa* mitogenome.

**Table S2:** Codon usage and relative synonymous codon usage (RSCU) of *A. puspa* mitogenome.

## Data Availability

The mitogenome has been deposited in the GenBank database under the accession number: PX797440.
