# Supplementary figures and images for: The First Complete Mitochondrial Genome of Common Hedge Blue Acytolepis puspa (Lepidoptera: Lycaenidae), and Comparative Genomic Analysis Within Polyommatinae
Source: Ecol Evol. 2026 Mar 29;16(4):e73326. doi: 10.1002/ece3.73326 (PMC13107287; doi:10.1002/ece3.73326)

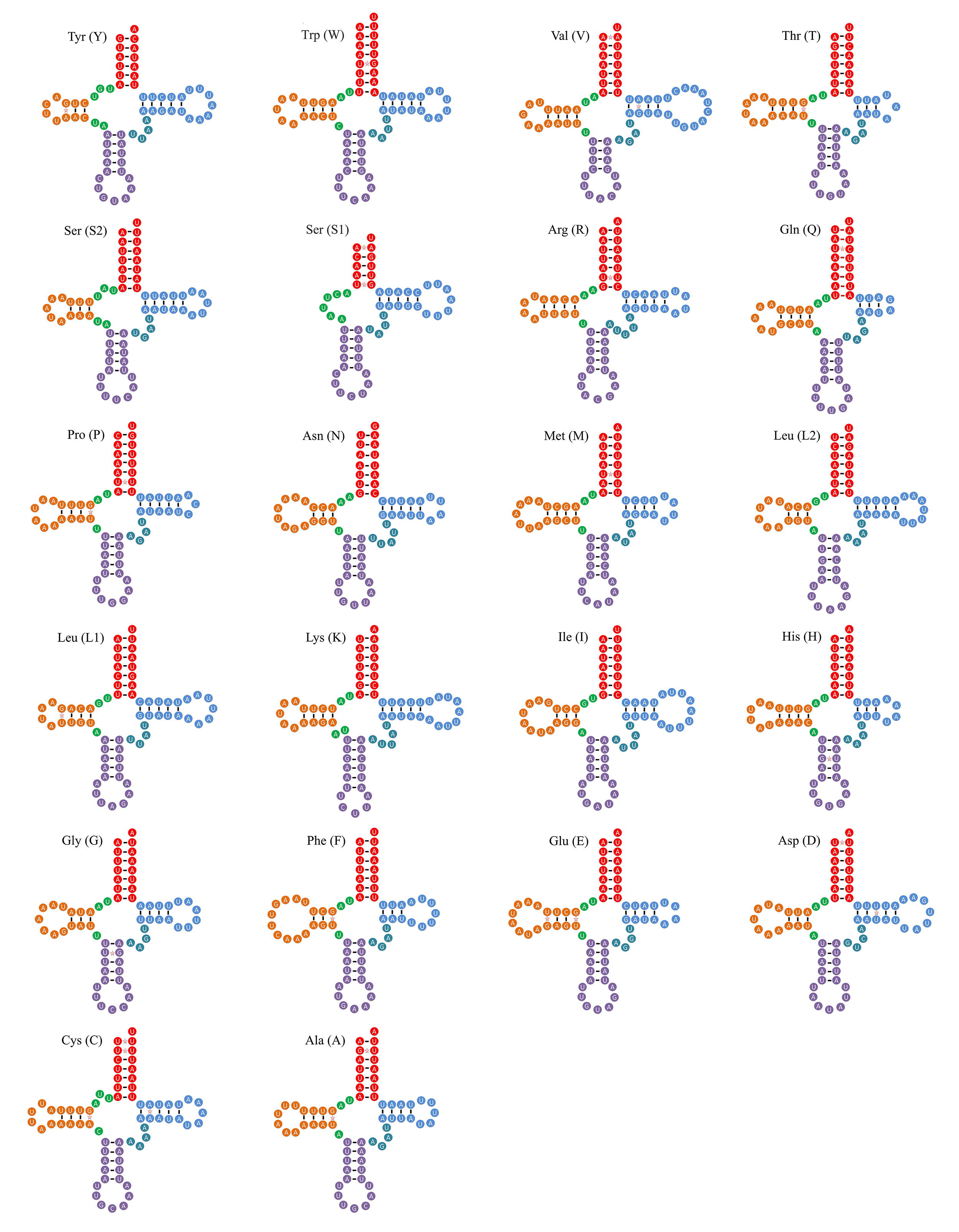

Supplement: Supplementary file 1 — Figure S1: Predicted secondary structures for the tRNAs of A. puspa mitogenome. Red: amino acid acceptor arm; Blue: TψC arm; Aqua green: variable loop; Purple: anticodon arm; Orange: dihydrouridine arm. [file ECE3-16-e73326-s004.jpg]

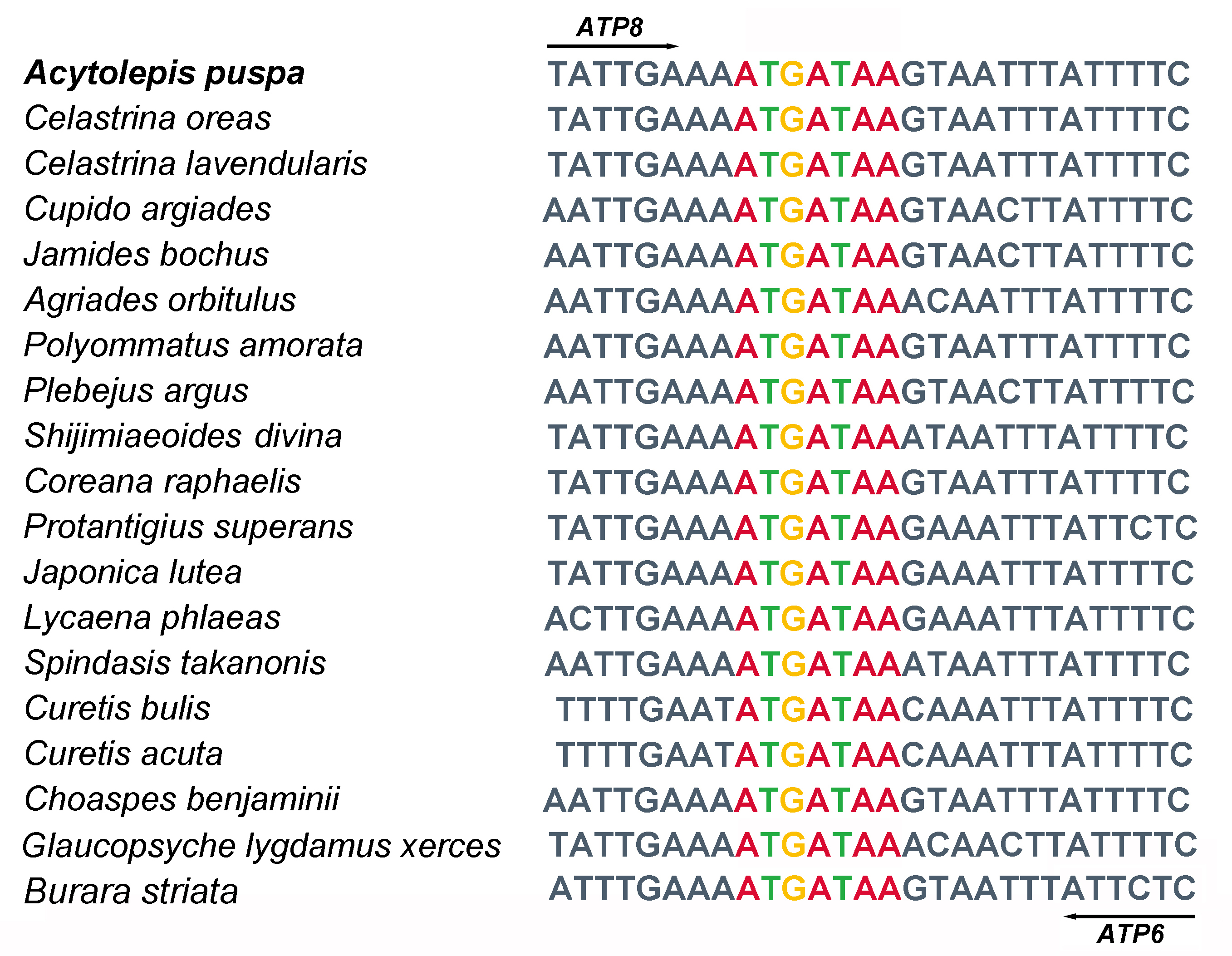

Supplement: Supplementary file 2 — Figure S2: The conserved overlapping sequences between ATP8 and ATP6 across lepidopterans used in this study. [file ECE3-16-e73326-s001.jpg]
