# Supplementary material for: The First Complete Mitochondrial Genome of Common Hedge Blue Acytolepis puspa (Lepidoptera: Lycaenidae), and Comparative Genomic Analysis Within Polyommatinae
Source: Ecol Evol. 2026 Mar 29;16(4):e73326. doi: 10.1002/ece3.73326 (PMC13107287; doi:10.1002/ece3.73326)
Supplement: Supplementary file 3 — Table S1: Nucleotide composition and AT/GC skew patterns in different regions of A. puspa mitogenome. [file ECE3-16-e73326-s003.docx]

**Table S1.** Nucleotide composition and AT/GC skew patterns in different regions of *A. puspa* mitogenome.

| **Regions** | **Size (bp)** | **A(%)** | **T(%)** | **C(%)** | **G(%)** | **AT(%)** | **GC(%)** | **AT-skew** | **GC-skew** |
| --- | --- | --- | --- | --- | --- | --- | --- | --- | --- |
| whole genome | 15,511 | 40.62 | 41.42 | 10.29 | 7.67 | 82.04 | 17.96 | −0.011 | −0.149 |
| PCGs | 11,165 | 34.90 | 45.96 | 9.07 | 10.07 | 80.86 | 19.14 | −0.137 | 0.052 |
| rRNAs | 2131 | 44.58 | 40.83 | 5.06 | 9.53 | 85.41 | 14.59 | 0.044 | 0.305 |
| tRNAs | 1465 | 41.77 | 41.09 | 7.17 | 9.97 | 82.86 | 17.14 | 0.008 | 0.163 |
| A+T-rich | 366 | 44.26 | 46.17 | 7.38 | 2.19 | 90.43 | 9.57 | −0.021 | −0.542 |
