# Supplementary material for: The First Complete Mitochondrial Genome of Common Hedge Blue Acytolepis puspa (Lepidoptera: Lycaenidae), and Comparative Genomic Analysis Within Polyommatinae
Source: Ecol Evol. 2026 Mar 29;16(4):e73326. doi: 10.1002/ece3.73326 (PMC13107287; doi:10.1002/ece3.73326)
Supplement: Supplementary file 4 — Table S2: Codon usage and relative synonymous codon usage (RSCU) of A. puspa mitogenome. [file ECE3-16-e73326-s002.docx]

**Table S2.** Codon usage and relative synonymous codon usage (RSCU) of *A. puspa* mitogenome.

| **Codon** | **Number** | **RSCU** | **Codon** | **Number** | **RSCU** | **Codon** | **Number** | **RSCU** | **Codon** | **Number** | **RSCU** |
| --- | --- | --- | --- | --- | --- | --- | --- | --- | --- | --- | --- |
| UUU(F) | 359 | 1.95 | UCU(S) | 87 | 2.06 | UAU(Y) | 183 | 1.87 | UGU(C) | 32 | 2 |
| UUC(F) | 10 | 0.05 | UCC(S) | 7 | 0.17 | UAC(Y) | 13 | 0.13 | UGC(C) | 0 | 0 |
| UUA(L) | 491 | 5.42 | UCA(S) | 125 | 2.95 | UAA(*) | 0 | 0 | UGA(W) | 94 | 3 |
| UUG(L) | 15 | 0.17 | UCG(S) | 0 | 0 | UAG(*) | 0 | 0 | UGG(W) | 1 | 1 |
| CUU(L) | 20 | 0.22 | CCU(P) | 66 | 2.3 | CAU(H) | 58 | 1.78 | CGU(R) | 9 | 0.43 |
| CUC(L) | 2 | 0.02 | CCC(P) | 3 | 0.1 | CAC(H) | 7 | 0.22 | CGC(R) | 1 | 0.05 |
| CUA(L) | 16 | 0.18 | CCA(P) | 45 | 1.57 | CAA(Q) | 57 | 1.93 | CGA(R) | 42 | 2.02 |
| CUG(L) | 0 | 0 | CCG(P) | 1 | 0.03 | CAG(Q) | 2 | 0.07 | CGG(R) | 1 | 0.05 |
| AUU(I) | 453 | 1.79 | ACU(T) | 63 | 1.87 | AAU(N) | 247 | 1.91 | AGU(S) | 34 | 0.8 |
| AUC(I) | 21 | 0.08 | ACC(T) | 9 | 0.27 | AAC(N) | 12 | 0.09 | AGC(S) | 1 | 0.02 |
| AUA(M) | 285 | 1.13 | ACA(T) | 63 | 1.87 | AAA(K) | 103 | 1.89 | AGA(S) | 72 | 3.46 |
| AUG(M) | 24 | 1 | ACG(T) | 0 | 0 | AAG(K) | 6 | 0.11 | AGG(S) | 0 | 0 |
| GUU(V) | 70 | 2.22 | GCU(A) | 65 | 2.28 | GAU(D) | 60 | 1.88 | GGU(G) | 75 | 1.54 |
| GUC(V) | 1 | 0.03 | GCC(A) | 6 | 0.21 | GAC(D) | 4 | 0.13 | GGC(G) | 2 | 0.04 |
| GUA(V) | 51 | 1.62 | GCA(A) | 42 | 1.47 | GAA(E) | 69 | 1.92 | GGA(G) | 107 | 2.19 |
| GUG(V) | 4 | 0.13 | GCG(A) | 1 | 0.04 | GAG(E) | 3 | 0.08 | GGG(G) | 11 | 0.23 |
